# Genetic mouse models of migraine

**DOI:** 10.1186/s10194-019-1029-5

**Published:** 2019-07-12

**Authors:** Daniela Pietrobon, K. C. Brennan

**Affiliations:** 1 Department of Biomedical Sciences and Padova Neuroscience Center, University of Padova, 35131 Padova, Italy; 2 CNR Institute of Neuroscience, 35131 Padova, Italy; 3 Department of Neurology, University of Utah, Salt Lake City, Utah USA

**Keywords:** Migraine, Cortical spreading depression, Genetic mouse model, Glutamate, Synaptic transmission, Trigeminovascular pain pathway

## Abstract

Mouse models of rare monogenic forms of migraine provide a unique experimental system to study the cellular and circuit mechanisms of the primary brain dysfunctions causing a migraine disorder. Here, we discuss the migraine-relevant phenotypes and the migraine-relevant functional alterations in the brain of five genetic mouse models of migraine, four of which carry mutations derived from patients with familial hemiplegic migraine (FHM) and the fifth carry a mutation from patients with both phenotypically normal MA and familial advanced sleep phase syndrome (FASPS). We focus on the latter mouse model, in which a ubiquitous serine-threonine kinase is mutated, and on two mouse models of pure FHM, in which a voltage-gated calcium channel controlling neurotransmitter release at most brain synapses and a Na/K ATPase that is expressed mainly in astrocytes in the adult brain are mutated, respectively. First, we describe the behavioral phenotypes of the genetic animal models and review the evidence that an increased susceptibility to experimentally induced cortical spreading depression (CSD) is a key migraine-relevant phenotype common to the five models. Second, we review the synaptic alterations in the cerebral cortex of the genetic models of migraine and discuss the mechanisms underlying their increased susceptibility to CSD. Third, we review the alterations in the trigeminovascular pain pathway and discuss possible implications for migraine pain mechanisms. Finally, we discuss the insights into migraine pathophysiology obtained from the genetic models of migraine, in particular regarding the mechanisms that make the brain of migraineurs susceptible to the ignition of “spontaneous” CSDs. Although the reviewed functional studies support the view of migraine as a disorder of the brain characterized by dysfunctional regulation of the excitatory/inhibitory balance in specific neuronal circuits, much work remains to be done in the genetic mouse models e.g. to identfy the relevant dysfunctional circuits and to establish whether and how the alterations in the function of specific circuits (in the cerebral cortex and/or other brain areas) are state-dependent and may, in certain conditions, favor CSD ignition and the migraine attack.

## Introduction

Migraine is much more than an episodic headache and a pain disorder. It is a complex brain disorder primarily affecting the sensory nervous system and characterized by a global dysfunction in multisensory information processing and integration. Indeed, in most attacks, the typical throbbing unilateral headache is associated with amplification of percepts from multiple senses indicating amplification of sensory gain. Hypersensitivity to sensory stimuli may persists in the interictal period, during which the brain of migraineurs show several alterations in sensory physiology. Interestingly the magnitude of some of these alterations increases in the interictal period towards the next attack and becomes maximal the day before the attack in temporal coincidence with prodromal symptoms (such as difficulty with speech, reading, concentration, increased emotionality, irritability, sensory hypersensitivity) that in many migraineurs are highly predictive of the attack [1–5]. The neurobiological mechanisms of the causative brain dysfunctions underlying the onset of a migraine attack and the alterations in multisensory information processing remain largely unknown and are key unanswered questions in migraine neurobiology.

In about 30% of migraineurs the headache is preceded by transient sensory (most frequently visual) disturbances, the so called migraine aura, whose neurophysiological correlate is now recognized to be cortical spreading depression (CSD) [6, 7]. CSD is a self-sustaining, slowly propagating (2–5 mm/min) wave of nearly complete depolarization of a sizable population of brain cells that lasts about one minute and silences brain electrical activity for several minutes. CSD can be induced in healthy brain tissue by intense depolarizing stimuli that increase the extracellular concentration of K^+^ ions, [K]_e_, above a critical threshold and release glutamate and other neurotransmitters. Although already the first studies of CSD mechanisms in the 50s pointed to [K]_e_ and glutamate as key players in the CSD initiation mechanisms, these mechanisms are still incompletely understood [6].

There is evidence from animal studies that CSD can activate and sensitize the trigeminovascular pain pathway and hence can initiate the headache mechanisms [2, 8–13]. It is generally believed that the migraine headache begins with the activation and sensitization of trigeminal sensory afferents, that innervate cranial tissues, in particular the meninges, and subsequent activation and sensitization of second order neurons in the trigeminocervical complex (comprising the trigeminal subnucleus caudalis and dorsal horn of the first cervical segments, indicted here for simplicity as TNC) and of higher order neurons in areas of the brainstem and forebrain to which the TNC projects directly or indirectly; these areas are involved in different aspects of pain and in the complex migraine symptomatology [1, 2, 4]. Whereas the properties of pial afferents remain largely unknown, the dural afferents are nociceptors with properties similar to those in the rest of the body [1, 14]. A sterile meningeal inflammation is considered to be a key mechanism that may underlie the sustained activation and sensitization of meningeal nociceptors during migraine attacks [1, 14].

It has been shown that a single experimental CSD can lead to delayed sustained increases in dural blood flow and in ongoing activity of rat dural nociceptors and TNC trigeminovascular neurons as well as delayed sensitization of these neurons [8–10, 12, 15]. It has been suggested that the delayed trigeminal activation may result from CSD-induced release of proinflammatory molecules in the meninges e.g. as a consequence of a parenchimal inflammation initiated by CSD-induced opening of pannexin1 channels and inflammasome activation [11] and/or as a consequence of CSD-induced pial and dural macrophage activation [16]. Activation of peptidergic meningeal nociceptors and consequent release of proinflammatory neuropeptides, including calcitonin gene-related peptide (CGRP), from their peripheral nerve endings can then further promote meningeal inflammation [1, 14]. Given the efficacy of monoclonal antibodies against CGRP in migraine treatment [17], it is interesting that intravenous administration of such antibodies inhibited the CSD-induced activation of A-δ meningeal nociceptors and the CSD-induced activation and sensitization of high threshold TNC neurons which receive input from A-δ fibers [12, 18]. Moreover, a CGRP receptor antagonist reversed CSD-induced behavioural alterations associated with pain perception in awake animals, such as freezing, grooming and reduced thresholds of tactile allodynia, without blocking CSD waves in the cortex [13]. Thus, understanding the neurobiological mechanisms that make the brain of migraineurs susceptible to ignition of spontaneous CSDs is another (or perhaps the) key unanswered question in migraine neurobiology.

Migraine is a complex polygenic genetic disorder, with heritability estimates as high as 50% [19, 20]. Although genome-wide association studies (GWAS) are providing increasing insights into the common genetic variants associated with migraine [21], the study of the functional consequences of GWAS hits is very difficult, if not impossible, given also the fact that they generally lie in intronic or intergenic regions and therefore they likely influence gene regulation rather than directly protein function. Thus, “common” migraine is not amenable to being instantiated in a mouse model and defies attempts at determining mechanism. In contrast, rare monogenic forms of migraine are caused by mutations that directly affect protein function, and the functional consequences of the disease-causing mutations can be studied in genetic mouse models of the disease. Thus far there are five monogenic migraine mutations that have knock-in (KI) mouse lines associated with them, allowing investigation of the underlying mechanisms. Four of these are derived from patients with familial hemiplegic migraine (FHM) and one from patients with both migraine with aura (MA) and familial advanced sleep phase syndrome (FASPS), a rare sleep condition in which individuals go to sleep unusually early in the evening and wake early in the morning.

Apart from the motor weakness or hemiplegia during aura and the possible longer duration of aura, typical FHM attacks resemble common MA attacks and both types of attacks may alternate in patients and co-occur within families [20, 22]. Thus, FHM and MA are considered to be part of the same spectrum and may share pathogenetic mechanisms, despite clinical observations that the response to infusion of CGRP and glycerylnitrate seem to differ [20] [23]. Some FHM patients can have “atypical” severe attacks and show additional ictal and/or permanent neurological features such as epilepsy, loss of consciousness, ataxia and cognitive impairment [20, 22].

Thus far, three FHM causative genes, all encoding ion channels or transporters, have been identified: CACNA1A (FHM1), ATP1A2 (FHM2) and SCNA1A (FHM3). CACNA1A and SCNA1A encode the pore-forming subunits of the voltage-gated ion channels Ca_V_2.1 and Na_V_1.1, while ATP1A2 encodes the α2 Na/K ATPase (α2 NKA) [24–26]. Ca_V_2.1 channels are widely expressed in the nervous system, including all structures implicated in the pathogenesis of migraine; being localized at the active zones of most brain synaptic terminals, they play a dominant role in initiating synaptic transmission, particularly at central synapses; their somatodendritic localization points to additional postsynaptic roles ( [27] and references therein). FHM1 mutations produce gain of function of recombinant human Ca_V_2.1 channels, mainly due to increased channel open probability and channel activation at lower voltages; the gain-of-function effect may be dependent on the specific Ca_V_2.1 splice variant and/or auxiliary subunit [28] ( [27] and references therein).

Being highly expressed in inhibitory interneurons in several brain areas and being mainly localized at the axon initial segment, Na_V_.1.1 channels play a key role in interneurons excitability, particularly in sustaining high-frequency firing [29–31]. Indeed loss-of-function mutations in Na_V_1.1 channels cause a spectrum of epilepsy syndromes [32]. Although the findings regarding the functional consequences of FHM3 mutations on recombinant human Na_V_1.1 channels are somewhat conflicting, overall they suggest that, most likely, FHM3 is associated with gain-of-function of Na_V_1.1 channels and consequent selective hyperexcitability of cortical interneurons [33].

The α2 NKAs are primarily expressed in neurons during development and at the time of birth and almost exclusively in astrocytes in the adult brain [34–38]. At cortical excitatory synapses the α2 NKAs are colocalized with GLAST and GLT-1 glutamate transporters at perisynaptic astrocytic process [35, 39], where a large fraction of GLT-1/α2 NKA couples exhibit a separation distance indicative of physical coupling [38], thus supporting a key role of α2 NKAs in glutamate clearance during synaptic activity [39]. In contrast, α2 NKAs are not present in the large majority of astrocytic processes surrounding inhibitory synapses [35, 38]. α2 NKAs play also an important role in K^+^ clearance during neuronal activity [39] [40] and, most likely, in astrocytic Na^+^ and Ca^2+^ homeostasis [41, 42]. FHM2 mutations cause the complete or partial loss-of-function of recombinant α2 NKAs [22, 43].

A mutation in the casein kinase 1δ (CK1δ) gene, which among many other functions serves as a circadian clock gene, was identified as a cause of FASPS in a family that presented for clinical evaluation of a debilitating MA and, in addition, exhibited circadian patterns consistent with FASPS [44, 45]. CKIδ is a ubiquitous serine-threonine kinase that phosphorylates the circadian clock protein Per2 and many other proteins involved in brain signaling [46]. The identified CK1δ mutation resulted in reduced enzyme function in vitro [45]. Later, a second family was identified, with a second mutation in the coding region of the CK1δ gene – in this family again both advanced sleep phase and migraine segregated with the mutation [47].

The FHM1, FHM2 and CK1δ mouse models of migraine, that are the subject of the present review, provide a unique experimental system to study the cellular and circuit mechanisms of the primary brain dysfunctions causing a migraine disorder, and thus to tackle the key unanswered questions in migraine neurobiology mentioned above.

### Genetic mouse models of migraine

Four different FHM mouse models were generated by introducing the human FHM1 R192Q or S218 L and FHM2 W887R or G301R mutations into the orthologous genes [48–51]. Whereas mutations R192Q and W887R cause in humans typical FHM attacks without additional clinical features (pure FHM) [24, 25], mutations S218 L and G301R cause severe clinical syndromes with atypical attacks that may include, in addition to hemiplegic migraine, prolonged coma/torpor or confusional state, epileptic seizures, elevated temperature, cerebral edema, transient or permanent cerebellar signs such as ataxia, nystagmus and cerebellar atrophy [52] [53, 54]*.* In agreement with the gain-of-function of FHM1 mutant human Ca_V_2.1 channels [27] and the loss-of-function of FHM2 mutant human α2 NKAs [43], an increased neuronal Ca^2+^ current was measured in FHM1 mice in different types of neurons [27, 48, 55] and the brain expression of the α2 NKA was about 50% reduced in heterozygous FHM2 mice [50, 51]. The more severe clinical phenotype caused by the S218 L FHM1 mutation correlates with the larger gain-of-function of recombinant human and native neuronal mouse Ca_V_2.1 channels produced by the S218 L compared to the R192Q mutation [49, 56]. In contrast, such correlation is not evident for the FHM2 mutations, since both the W887R and the G301R mutations completely eliminate pump activity of recombinant human α2 NKAs [57, 58] and reduce to a similar extent the α2 expression in the brain of adult heterozygous W887R and G301R mice [50, 51], likely due to ER retention and degradation of the misfolded protein [50, 54] (but cf. [59] for unaltered expression of the α2 NKA in the hippocampus of G301R mice in contrast with [51]).

The CK1δ mouse model was generated by inserting the human T44A mutation into the mouse genome via bacterial artificial chromosome (BAC) technique [47]. The migraines in patients with the T44A CK1δ mutation are phenotypically normal (they do not involve hemiplegia) [47].

We will focus on reviewing the functional studies in FHM1 and FHM2 KI mice which carry mutations causing pure FHM and thus should model migraine as close as possible, and will discuss the insights into migraine pathophysiology obtained from these studies. We will only briefly discuss the different findings in FHM1 and FHM2 KI mice which carry the severe syndrome-causing mutations and might give insights into the additional clinical features associated with these mutations. For the CK1δ mouse, as the subjects’ migraines are phenotypically normal, all migraine relevant phenotypes may be useful to extrapolate more generally to the migraine population; however for this model we need to consider whether the association with a sleep disorder affects the insights generated.

### Migraine-relevant phenotypes in the genetic mouse models

#### Behavioural phenotypes

Homozygous KI mice carrying the R192Q pure FHM1 mutation (FHM1 mice) and heterozygous KI mice carrying the W887R pure FHM2 mutation (FHM2 mice) do not show an overt phenotype [48, 50]. However, the FHM1 mice showed signs of photophobia in a modified elevated plus maze in which the safe closed arms were brightly illuminated. Moreover, behavioral changes suggestive of unilateral head pain, such as increased head grooming, abnormal eye blinking, sustained single-eye closures and full-body shuddering, were reported in FHM1 mice when subjected to novelty or restrain stress [60]. Interestingly, systemic administration of the antimigraine drug rizatriptan normalized these pain measures. Given the higher female prevalence in migraine, another interesting finding is that the increase in shudder/blink frequency was larger in female mice [60]. These kind of data are not yet available for the FHM2 and CK1δ mice. However, in a SHIRPA primary screening assessing sensory, motor and neuropsychiatric functions, the FHM2 mice showed an increased level of fear/anxiety as the only behavioural anomaly [50], a feature that does not appear to be shared by the FHM1 mice [60].

Heterozygous KI mice carrying the severe G301R FHM2 mutation (G301R FHM2 mice) revealed several behavioural alterations, which although not comprising altered level of anxiety, included increased startle response to aversive acoustic stimuli, stress-induced depression-like phenotypes, decreased sociability and increased compulsive behaviour (as shown by increased face and body grooming and increased marble-burying); the latter was female-specific and reverted by progestin-only contraceptive treatment [51]. However, these symptoms were not reported in the two families in which the G301R mutation was identified (except for obsessive compulsive disorder in one member) [53, 54] and, with the exception of the hypersensitivity to noxious stimuli and, possibly, the stress-induced depression-like phenotypes, they are not part of the core symptoms reported for either FHM or migraine.

Homozygous KI mice carrying the severe S218L FHM1 mutation (S218L FHM1 mice) exhibit the main features of the human S218 L clinical syndrome, including attacks of hemiparesis, attacks of generalized seizures, mild cerebellar ataxia and brain edema after mild head impact. These features were not observed in heterozygous S218L mice [49].

#### Cortical spreading depression

A key migraine-relevant phenotype that the four FHM animal models have in common is increased susceptibility to experimentally induced CSD, as revealed by a lower stimulation threshold for initiation of CSD in vivo and in vitro [39, 48–50, 55] or a higher frequency of CSDs elicited by prolonged epidural high KCl application in vivo [61, 62]. Moreover, the rate of CSD propagation was increased in all four FHM animal models.

The CK1δ model provides additional evidence for increased CSD susceptibility as a key phenotype of models of MA. CK1δ mice had a decreased CSD initiation threshold as well as an increased number of CSD to a constant stimulus. There was a trend toward faster CSD velocity in mutants compared to wild type littermates, but this was not significant. [47].

In agreement with the higher female prevalence in migraine, the velocity of propagation and the frequency of CSDs induced by prolonged KCl were larger in female than in male in both FHM1 mouse mutants; the sex difference was abrogated by ovariectomy and enhanced by orchiectomy, suggesting that female and male gonadal hormones exert reciprocal effects on CSD susceptibility [61, 63]. However, no gender differences in induction and propagation of experimental CSD were found in FHM2 KI mice [50, 62], although, interestingly, the CSD frequency induced by prolonged KCl in postmenopausal female G301R FHM2 mutants was lower than in aged males and younger females [62], suggesting that perhaps a gender-based difference might be present in a limited period of the female menstrual cycle. In the CK1δ mouse, there was an intermediate sex phenotype between the FHM1 and FHM2 models. There was a gradient of CSD thresholds, with increasing threshold in the order mutant female < mutant male < wild type female < wild type male. Taken overall, the difference in thresholds between the four groups was significant, but on post-hoc testing this difference appeared to be driven by the difference between the two most separated groups (mutant female and wild type male). These experiments were not powered to detect a sex difference, so future work may confirm or refute the phenotype more definitively [47].

In male FHM1 mice, the frequency of CSDs induced by prolonged KCl was also increased after administration of the stress hormone corticosterone, but not after acute restrain stress [64]. Neither the stress hormone nor restrain stress affected CSD frequency in male wild type mice [64]. Even chronic stress did not affect CSD frequency and velocity in male wild type mice [65, 66]; however, interestingly, the threshold for CSD induction was lower after either acute or chronic stress [66], pointing to the possibility that the difference in CSD threshold between FHM mutants and wild type mice might be larger in stressed compared to control animals. Measurements of CSD threshold in FHM mutants after stress remain to be done.

In contrast with wild type mice, a single CSD caused prolonged hemiplegia with leaning and circling in FHM1 mice, and CSD readily propagated into the striatum in the majority of FHM1 but not wild type mice, suggesting corticostriatal CSD propagation as a likely explanation for the more severe motor deficits in FHM1 mutants [61, 67, 68].

Moreover, the typical reduction of cerebral blood flow after CSD was more prolonged in FHM1 mice [69] and more severe in heterozygous S218L FHM1 mice [70]. In contrast, the cerebral blood flow in G301R FHM2 mice was similar to that in wild type mice, but the typical reduction of spontaneous activity after CSD was more prolonged [51].

During CSD, the increase of the intracellular concentration of Ca^2+^ ions [Ca^2+^]_in_ in cortical neurons and neuropil was larger in both FHM1 mouse models compared to wild type mice [69, 70], which was expected given the gain-of-function of Ca_V_2.1 channels in cortical pyramidal cells from both mutants [55, 71]. Consistent with this, the metabolic oxygen consumption was larger in FHM1 mice and resulted in a larger decrease of tissue oxygenation during CSD compared to wild type mice, due to a larger mismatch between oxygen consumption and oxygen supply from the cerebral blood flow (CBF), being CBF similar in the two genotypes during CSD [69] (but cf. [70]).

In good correlation with the larger Ca_V_2.1 gain-of-function produced by the S218L compared to the R192Q mutation [28, 48, 49, 56], the strength of CSD facilitation as well as the severity of the post-CSD neurological motor deficits and the propensity of CSD to propagate into subcortical structures were larger in S218L compared to R192Q FHM1 mice [49, 61, 67, 68]. Moreover, unlike the R192Q mice, the S218 L mice frequently developed multiple CSDs after a single CSD-inducing stimulus, which were more frequent in homozygotes compared to heterozygotes [49]. Furthermore, unlike the R192Q mice (and the heterozygous S218L), the homozygous S218L mutants developed generalized seizure after about 1 h from a single CSD [61]. In awake G301R FHM2 mice, full tonic-clonic seizures were frequently observed after a certain number of CSDs elicited by continous KCl application [62]. These unique CSD features might contribute to the additional clinical symptoms in the severe human syndromes caused by the S218L and G301R mutations.

### Migraine-relevant alterations in the brain of genetic mouse models of migraine

#### Synaptic alterations in the cerebral cortex and CSD mechanisms

Direct measurements of synaptic transmission at different cortical synapses in pure FHM1 mice invariably revealed enhanced glutamatergic transmission at the excitatory synapses, due to enhanced action-potential (AP) evoked Ca^2+^ influx through mutant presynaptic Ca_V_2.1 channels and enhanced probability of glutamate release [55]. As expected from the enhanced release probability, short-term depression was increased at mutant excitatory synapses on both pyramidal cells and fast-spiking interneurons (athough to a different extent) [55]. In striking contrast with glutamatergic transmission, GABAergic transmission at different cortical inhibitory synapses was unaltered in FHM1 mice, despite being initiated by Ca_V_2.1 channels [55, 72]. The lack of effect on inhibitory synaptic transmission in contrast with the gain-of-function effect on excitatory transmission appears to be a common feature of FHM1 mutations since it was shown also for the severe S218L mutation [71]. Expression of interneuron-specific Ca_V_2.1 channels whose gating properties are barely affected by the FHM1 mutation likely underlies this unexpected finding [72].

Although inhibitory transmission was not investigated, evidence for increased glutamatergic neurotransmission in FHM1 mice was also obtained at several other excitatory synapses in different brain areas, including those onto dorsal suprachiamastic nucleus neurons [73], cerebellar parallel fibers-Purkinje cell synapses [74] and stratum radiatum CA1 synapses [75]. Interestingly, long-term potentiation (LTP) at these hippocampal synapses was enhanced in FHM1 mice, while long term depression was unaltered; paradoxically, spatial learning in contextual fear conditioning and Morris water maze tests was impaired [75].

In the cerebral cortex, specialized core microcircuits, which involve different types of inhibitory interneurons and mediate feedback inhibition, feedforward inhibition and disinhibition, regulate the relative strenght of excitatory and inhibitory synaptic conductances in cortical neurons and their temporal and spatial relationships and, thus, dynamically maintain the excitatory-inhibitory (E/I) balance necessary for the transfer of information while preventing runaway excitation [76]. These microcircuits are essential for the correct processing of sensory information (e.g. for sensory gain control, surround suppression, synchronization and generation of cortical rythms, formation of cell assemblies and information transfer to higher areas) [76, 77].

The differential effect of FHM1 mutations on excitatory and inhibitory synaptic transmission (and on short-term synaptic plasticity at different cortical synapses) implies that these core microcircuits are most likely dysfunctional in FHM1 [78] and points to impaired regulation of cortical E/I balance as a primary brain dysfunction and a key pathogenic mechanism in FHM1 [79]. On the basis of the available data on the effect of the FHM1 mutations on synaptic transmission at different cortical synapses, it is not straightforward to predict their effect on microcircuit and network function. In fact, while enhanced excitatory transmission at the synapses on cortical principal neurons would increase network excitation, enhanced transmission at the synapses onto inhibitory interneurons may lead to increased recruitment of the interneurons and hence to increased inhibition.. This could, at least in part, explain the unexpected reduction in neuronal calcium responses to prolonged repeated whisker stimulation reported in FHM1 mice [69].

As shown by CSD rescue experiments, there is a causative link between increased glutamatergic transmission at cortical synapses and facilitation of experimental CSD in FHM1 mice. In fact, the facilitation of initiation and propagation of CSD in mutant cortical slices was completely eliminated when AP-evoked glutamate release at pyramidal cell synapses was brought back to wild type values by partially inhibiting the Ca_V_2.1 channels [55]. The finding that propagation of CSD to subcortical structures in FHM1 mice was eliminated by systemic treatment with pregabalin, a drug that reduced excitatory transmission in mutant hippocampal slices, suggests that the increased propensity of CSD to propagate into subcortical structures is also linked to increased excitatory neurotransmission [68].

The important role of excessive glutamatergic transmission in migraine mechanisms, in particular CSD susceptibility, is underscored and supported by the functional studies in pure FHM2 mice. These mutants show reduced rate of synaptic glutamate clearance by cortical astrocytes during neuronal activity and reduced density of glutamate transporters GLT-1 at perisynaptic astrocytic processes, which mirrors the reduced expression of the α2 NKA [39].

LTP induced by high frequency stimulation at the hippocampal perforant path synapses in the dentate gyrus was enhanced in the FHM2 mice; in contrast, LTP was unaltered at stratum radiatum-CA1 synapses [80]. Basal synaptic transmission was unaltered in both areas, judging from the similar input-output curves and paired pulse ratios in field recordings in wild type and mutant hippocampal slices [80]. These findings may be consistent with the larger impairment of glutamate clearance uncovered in FHM2 cortical slices after high-frequency stimulation of glutamate release compared to low frequency (single pulse) stimulation [39]. Also consistent with this is the finding that mice with a 60–80% reduction of GLT-1 expression (after conditional knockout in adolescents) show unaltered basal synaptic transmission at corticostriatal synapses, but decreased EPSC depression during prolonged stimulation [81]. Interestingly, these mice showed increased compulsive behaviour (as shown by increased self-grooming), which was rescued by treatment with the NMDA glutamate receptor (NMDAR) antagonist memantine.

Memantine treatment also rescued the female-specific compulsive behaviour in heterozygous G301R FHM2 mice [51], suggesting that, although not directly measured, also in these mice the expression of GLT-1 receptors is reduced (possibly more than in pure FHM2 mice which did not show compulsive behaviour) and results in impaired clearance of glutamate at excitatory synapses and increased NMDAR activation likely as a consequence of glutamate spillover. In apparent conflict with this, the rate of glutamate uptake measured in mixed neuron-astrocytes cultures from heterozygous G301R FHM2 embrios was not significantly decreased [51]*.* The unsuitability of astrocytic cultures for the study of NKA function [59] might underlie this finding.

Since, in the cortex, the α_2_ NKA pump is localized in astrocytic processes surrounding glutamatergic synapses and only in a small fraction, if any, of astrocytic processes surrounding GABAergic synapses [35, 38], FHM2 mutations likely affect excitatory but not inhibitory synaptic transmission, and thus may lead to altered circuit function and impaired regulation of cortical E/I balance, as in FHM1.

Interestingly, it has been shown that the defective glutamate clearance at cortical excitatory synapses in the FHM2 mice may largely account for the lower threshold for induction of experimental CSD in these mice [39]. The FHM2 mutants also showed reduced rate of K^+^ clearance during neuronal activity, and the defective clearance of both glutamate and K^+^ likely accounts for the increased rate of CSD propagation [39].

Overall the findings in the mouse models of pure FHM support the conclusion that their increased susceptibility to experimental CSD is largely due to excessive cortical glutamatergic transmission, arising from either increased glutamate release (FHM1) or impaired glutamate clearance (FHM2).

Together with pharmacological data in wild type mice providing strong support for a key role of glutamate NMDARs and Ca_V_2.1 channels in initiation and/or propagation of experimental CSD [6, 82, 83], the findings in FHM mice support a model of CSD initiation in which i) Ca_V_2.1-dependent glutamate release and consequent activation of NMDARs are key elements for generation of the net self-sustaining inward current necessary to initiate the positive feedback cycle that ignites a propagating CSD when the removal of K^+^ and glutamate from the interstitium does not keep pace with their release and ii) the α_2_ NKA pumps exert a dampening role owing to their key role in K^+^ and, in particular, glutamate clearance by astrocytes [6, 39, 55, 84]. Moreover, the findings are consistent with a model of CSD propagation in which interstitial K^+^ diffusion initiates the CSD positive-feedback cycle in contiguous dendritic regions [6, 39, 55, 85].

In FHM1 mice carrying the severe S218L mutation, gain-of-function of additional Ca_V_2.1-dependent processes, besides enhanced glutamatergic synaptic transmission, likely underlie the particularly high susceptibility to CSD and high propensity of CSD to spread into subcortical structures as well as some of the unique features of CSD, including its recurrence, which were not observed in pure FHM1 mice [49, 61, 67, 68, 71]. A specific feature of mice carrying the S218L mutation is the presence of a fraction of mutant Ca_V_2.1 channels that is open at resting potential, as revealed by the reduced frequency of miniature excitatory postsynaptic currents (mEPSCs) after block of Ca_V_2.1 channels in cortical slices from both heterozygous and homozygous S218L mutants [71], and, by increased baseline [Ca^2+^]_in_ in layer 2/3 axonal boutons and shafts in heterozygous S218L mice in vivo [70]. Both a reduction of mEPSCs frequency after blocking Ca_V_2.1 channels and an increase in basal [Ca^2+^]_in_ in synaptic terminals were measured at Calyx of Held synapses in brainstem slices from S218L mice [86]. In contrast, the mEPSCs frequency at cortical and brainstem synapses was not altered in pure FHM1 mice, indicating that presynaptic Ca_V_2.1 channels carrying the R192Q mutation are closed at resting potential in brain slices [55, 87]. Probably as a consequence of the increase in baseline [Ca^2+^]_in_, the heterozygous S218L mice showed some alterations in axonal and dendritic morphology in the resting state, including slightly larger boutons [88]. It remains to be seen whether this is a specific functional consequence of severe FHM1 mutations, which contributes to some of the additional clinical features associated with them.

#### Alterations in the trigeminovascular pain pathway and pain mechanisms

The function of the trigeminovascular (TGV) pain pathway is expected to be altered in FHM1 mice because Ca_V_2.1 channels are involved in controlling neurotransmitter release at different levels in the trigeminovascular system, e.g. CGRP release from capsaicin-sensitive perivascular terminals of meningeal nociceptors as well as release at central synapses onto TNC neurons including synapses of the descending inhibitory and facilitatory pathways that regulate TGV pain transmission ( [27] and references therein). However, relatively few studies investigated the function of the trigeminovascular pain network in FHM1 KI mice; moreover, most of these studies were in vitro and focused on the peripheral part of the network.

Investigation of CGRP release from dura mater in fluid-filled hemisected skulls revealed that neither basal nor K^+^-evoked CGRP release were significantly altered in adult FHM1 mice [89, 90]. Since a large fraction of peptidergic dural trigeminal ganglion (TG) afferents are capsaicin-sensitive [1], these findings are consistent with the fact that the Ca_V_2.1 current was unaltered in small capsaicin-sensitive TG neurons from adult FHM1 mice, which, according to retrograde labelling from the dura, constitute the majority of small dural afferents [89]. This may also contribute to explain the finding that dural artery vasodilation induced in vivo by systemic capsaicin was not increased in FHM1 mice; actually, vasodilation induced by both systemic capsaicin and CGRP was decreased [90], suggesting downregulation and/or desensitization of blood vessels CGRP receptors, perhaps as a compensatory mechanism. The lower fraction of CGRP-expressing neurons uncovered in trigeminal ganglia of FHM1 mice [91] may be an additional compensatory mechanism, that might also contribute to the unaltered CGRP release from dura mater in the FHM1 mutants.

All together, these findings argue against the idea that increased CGRP release from perivascular TG fibers at the dura and consequent increased vasodilation and mast cell degranulation facilitate the development of neurogenic inflammation (following activation of meningeal nociceptors e.g. by CSD) in FHM1 compared to wild type mice. Perhaps this is a consequence of compensatory mechanisms that might be triggered by the occurrence of spontaneous CSDs in the FHM1 mutants.

Judging from the finding of unaltered *c-fos* expression in the TNC after in vivo electrical stimulation of the dura in FHM1 mice [92], also synaptic transmission at the central terminals of dural TG afferents might be unaffected by the FHM1 mutation. This would be consistent with unaltered Ca_V_2.1 current in most dural TG afferents of FHM1 mice, as was shown in small capsaicin-sensitive dural afferents [89]. This further underscores the importance of neuron subtype-specific effects of FHM1 mutations in the FHM1 pathophysiology. Indeed, the finding of increased *c-fos* expression in several thalamic nuclei after in vivo electrical stimulation of the dura [92], is consistent with increased synaptic transmission at TNC-thalamus excitatory synapses as a consequence of gain-of-function of Ca_V_2.1 channels located at thalamic synaptic terminals of TNC neurons. Although this remains to be demonstrated, it would contribute to increase the gain of the TGV pain pathway in FHM1.

Depending on the study, K^+^-evoked CGRP release from isolated trigeminal ganglia was either increased [89] or unaltered [90] in adult FHM1 mice; in the latter study, also CGRP release from TNC was unaltered in the mutants. Enhanced K^+^-evoked CGRP release from trigeminal ganglia implies gain-of-function of Ca_V_2.1 channels in some TG neurons in FHM1 mice; this was indeed shown in a subpopulation of small capsaicin-insensitive neurons, which do not innervate the dura [89]. Given that in these neurons the action potential-evoked Ca_V_2.1 current is larger in FHM1 mice [89], one predicts enhanced transmitter release upon their activation. However, the function, transmitters and possible involvement in migraine pain of this subpopulation of small TG neurons remain unknown.

In cultured TG neurons from FHM1 mice pups, also basal (besides K^+^-evoked) CGRP release was increased, suggesting opening of mutant Ca_V_2.1 channels at resting potential [93]. Congruently, these cultured TG neurons show interesting Ca_V_2.1-dependent alterations such as loss of constitutive inhibition of ATP-gated P2X3 receptors (P2X3Rs) by brain natriuretic peptide receptors, which leads to increased P2X3R current and enhanced excitability in response to ATP in FHM1 mice [94–96]. The neuronal upregulation of the P2X3R function (as well as the upregulation of P2X7 receptors function recently uncovered in satellite glial cells and macrophages) were eliminated after inhibition of the CGRP receptors [93, 97, 98]. This is consistent with the idea that the increased basal release of CGRP promotes sensitization of P2X3R-expressing TG neurons, cross-talk between neurons and satellite glial cells and macrophages, resulting in a local persistent inflammatory environment in the FHM1 TG [93, 97, 98]. However, basal release of CGRP was not increased in trigeminal ganglia from adult FHM1 mice [89, 90], suggesting caution in drawing conclusions regarding migraine pain mechanisms from findings in pups TG cultures. Whether the adult TG shows a basal inflammatory phenotype in FHM1 mutants remains unclear, since in FHM1 ganglia the number of active macrophages was increased (in all divisions), but the protein level of the pro-inflammatory cytokines IL1beta, IL6 and TNFalpha was unaltered [99]. Interestingly, a larger fraction of TG neurons was immunoreactive for active phosphorylated CaMKII in FHM1 compared to wild type ganglia; the difference in amount of phosphoprotein between the two genotypes was eliminated after blockade of Ca_V_2.1 channels, suggesting facilitation of basal Ca_V_2.1-dependent Ca signalling in FHM1 TG neurons [94].

The CK1δ mouse showed strong evidence for activation of migraine-relevant pain pathways. Nitroglycerin (NTG) infusion has been used as a trigger for migraine without aura in humans [100], and was later adapted for rat [101]. After adaptation of the methods for mouse [102], it was used to test both heat and mechanical withdrawal thresholds in CK1δ mice and wild type littermates. There was a significant reduction in both heat and mechanical withdrawal thresholds in CK1δ mutant mice [47]. As NTG-induced threshold changes were responsive to the migraine abortive sumatriptan in wild type mice [102] these data were taken as evidence for an enhanced algesic response to a migraine trigger in CK1δ mice [47]. There was also a significant increase in the number of c-fos-reactive cells in the TNC after NTG in CK1δ mutant compared to wild type mice, consistent with increased activation of craniofacial pain networks by this migraine trigger [47].

#### Insights into migraine pathophysiology

The genetic mouse models of migraine support the view of migraine as a disorder of the brain characterized by dysfunctional regulation of the E/I balance in specific neuronal circuits in the cerebral cortex and other brain structures. Moreover, they support a key role of CSD in the pathogenesis of migraine with aura and provide insights into how a “spontaneous” CSD can arise in the brain of migraineurs.

The induction of experimental CSD in healthy tissue requires intense depolarizing stimuli that, according to the model of CSD initiation proposed in the previous section, increase the extracellular [K+] above a critical value and release enough glutamate to overwhelm the binding capacity of astrocytic glutamate transporters, thus leading to cooperative activation of the high number of synaptic and extrasynaptic NMDARs necessary for initiation of the CSD positive feedback cycle. In migraineurs CSD is not induced by experimental depolarizing stimuli, but arises “spontaneously” in certain conditions. How can this occurs?

The findings in the FHM mouse models suggest that ignition of a “spontaneous” CSD could be favoured by conditions leading to excessive activation of synaptic and extrasynaptic NMDARs, i.e. conditions leading to membrane depolarization and to overwhelming of the transport capacity of astrocytic glutamate transporters. This would probably require high-frequency repetitive or synchronous activity of a sufficient number of excitatory synapses in which glutamatergic transmission is potentiated, e.g. as a consequence of increased glutamate release as in FHM1 or reduced expression of astrocytic α2 NKA and glutamate transporters, as in FHM2, or as a consequence of other mechanisms in common migraine. We hypothesize that this may occur in certain conditions as a consequence of dysfunctional regulation of the E/I balance in specific cortical circuits. Much work remains to be done in the FHM models to identify the relevant dysfunctional cortical circuits and establish whether indeed dysfunctional regulation of the E/I balance in these circuits may favour CSD ignition and identify the specific conditions (brain states) in which this may occur.

The behavioural phenotypes and the functional analysis of the genetic mouse models of migraine are consistent with the concept of migraine as a disorder of sensory network gain and plasticity [3]. Much work remains to be done to investigate possible alterations in sensory processing in awake animals, that may underlie some of the interictal alterations in sensory processing shown by migraineurs, and to investigate the underlying cellular and circuit mechanisms. It will be also important to investigate whether the alterations in the function of specific circuits (in the cortex and/or other brain structures) in the genetic models are modulated by state-dependent changes in plasticity, and may thus underlie some of the interictal cyclical changes in sensory physiology and/or some of the prodromal symptoms shown by migraineurs.

#### Translational relevance

An important point to make at the outset is that all models are what their name says – models. As such they are abstractions from disease reality that allow cleaner testing of hypotheses than the disease state allows; or asking of questions that cannot even be asked in the disease state. In return for the abstraction and simplification from the disease state, models are ‘expected’ to generate insight that is not otherwise possible. In this regard all of the models described have both elements of simplification and abstraction; but they also have delivered on their promise. Probably the most important insight coming from the diverse genetic models of migraine is the role of circuit excitability, with CSD as the primary circuit phenotype, underlain perhaps by excessive glutamatergic neurotransmission and/or excessive activation of glutamate NMDARs.

The question arises whether models derived from rare monogenic forms of migraine can yield insights for the rest of the disease. Most migraineurs do not have hemiplegic migraine or any monogenic form; indeed most migraineurs do not have migraine with aura, and all models thus far come from families with migraine with aura. A first reply is that across biology, the use of rare mutants has enabled major discoveries about disease mechanisms relevant to the larger population. *Their value comes from their rarity*; their monogenic nature allows for identification of *specific mechanisms that would be impossible to detect for migraine in the larger population*. As to whether the insights gained from monogenic models are generalizable to migraine at large, the jury is still out, but there is reason to be very optimistic. The fact that diverse mutations in neurons and non-neuronal cells converge on a single circuit phenotype – CSD – is quite promising from the point of view of generalization. This is especially true for migraine with aura of course. It is also worth noting that the addition of the CK1δ mouse, from a family with *non-hemiplegic* migraine, strongly suggests that the insights gained from monogenic models are not confined to humans with hemiplegic migraine.

For the clinician who desires a ‘bottom line’ on the relevance of these genetic models of migraine, the most important point is that by allowing the pursuit of precise mechanisms (what protein is acting, in what way, on what circuit?) they also allow the pursuit of precise solutions – drugs, biologics, stimulation paradigms, or other ways of addressing the disease that we have not yet considered. They are not the only way to derive new treatments – brute-force empiricism has worked for centuries – but they are certainly more targeted, and arguably much more satisfying, because they have the ability to actually tell us how the disease works.

## Conclusions

Mouse models of rare monogenic forms of migraine provide a unique experimental system to study the cellular and circuit mechanisms of the primary brain dysfunctions causing a migraine disorder. A key migraine-relevant phenotype that these animal models have in common is increased susceptibility to experimentally induced CSD. In the FHM mouse models this is largely due to excessive cortical glutamatergic transmission, arising from either increased glutamate release (FHM1) or impaired glutamate clearance (FHM2). The genetic animal models provide insights into how a “spontaneous” CSD can arise in the brain of migraineurs and support the view of migraine as a disorder of the brain characterized by dysfunctional regulation of the E/I balance in specific neuronal circuits in the cerebral cortex and other brain structures. Much work remains to be done in these models to identify the relevant dysfunctional circuits and to establish whether and how the alterations in the function of specific circuits are state-dependent and may, in certain conditions, favour CSD ignition and the migraine attack.

## Data Availability

Not applicable.

## Abbreviations

[Ca^2+^]_in_: Intracellular Ca^2+^ concentration
[K]_e_: Extracellular concentration of K^+^ ions
AP: Action potential
1δ (CK1δ): Casein kinase
CBF: cerebral blood flow
CGRP: Calcitonin gene-related peptide
CSD: Cortical spreading depression
E/I: Excitatory-inhibitory balance
FASPS: Familial advanced sleep phase syndrome
FHM: Familial hemiplegic migraine
FHM1 mice: Homozygous knock-in mice carrying the R192Q pure FHM1 mutation
FHM2 mice: Heterozygous knock-in mice carrying the W887R pure FHM2 mutation
GWAS: Genome-wide association studies
KI: Knock-in
mEPSC: miniature excitatory postsynaptic current
NKA: Na/K ATPase
NMDAR: NMDA glutamate receptor
NTG: Nitroglycerin
P2X3R: P2X3 receptor
TG: Trigeminal ganglion
TGV: Trigeminovascular
TNC: Trigeminocervical complex
